# Dopant‐Tailored Matrices: A Crystal Engineering Strategy for Organic Room‐Temperature Phosphorescent Host–Guest Systems and Beyond

**DOI:** 10.1002/anie.8252460

**Published:** 2026-08-12

**Authors:** Catherine Demangeat, Raphael Rullan, Yipeng Tang, Bin Hu, Anthony D'Aléo, Tangui Le Bahers, Mohammad Esmail Alikhani, André‐Jean Attias

**Affiliations:** ^1^ Building Blocks for FUture Electronics Laboratory IRL 2002 CNRS‐Sorbonne Université‐Yonsei University Yonsei University Seoul Republic of Korea; ^2^ Department of Engineering Science The University of Electro‐Communications Tokyo Japan; ^3^ ENS de Lyon CNRS Lyon France; ^4^ State Key Laboratory of Luminescent Materials and Devices South China University of Technology Guangzhou P. R. China; ^5^ Institut de Physique et Chimie des Matériaux de Strasbourg (IPCMS) UMR 7504 CNRS‐Université de Strasbourg Strasbourg France; ^6^ MONARIS ‐ UMR 8233 Sorbonne Université ‐ CNRS Paris France

**Keywords:** crystal engineering, host–guest doped single‐crystals, organic room‐temperature phosphorescence, organic semiconductor single‐crystals

## Abstract

Host–guest doping of molecular crystals is a powerful strategy to tune optoelectronic properties, yet achieving precise host–dopant compatibility and beneficial synergy without introducing detrimental effects caused by dopant‐induced disorders remains challenging. Here, a new crystal engineering strategy is introduced in which host matrices are rationally designed to accommodate a predefined class of dopants and promote favorable host–guest interactions. This tailored‐dopant matrix concept is demonstrated for dopant‐induced organic room‐temperature phosphorescence (RTP) using carbazole‐based matrices and benzoindole‐based dopants, a prototypical RTP system. Guided by the hypothesis that a herringbone packing of carbazole units promotes synergistic structural interactions with the dopant, a multiscale theoretical methodology is first developed to elucidate the intermolecular interactions stabilizing this motif in pristine carbazole‐based crystals. These insights enable the design and synthesis of new host architectures exhibiting the targeted packing arrangement. Ultimately, the resulting single‐crystalline host–guest materials exhibit long‐lived organic RTP, with phosphorescence lifetimes of several hundred milliseconds. This work could establish dopant‐tailored crystal engineering as a potential new paradigm for designing functional doped organic semiconductor crystals with tailored optoelectronic properties.

## Graphical abstract

Rather than selecting dopants to match a pre‐existing host matrix, the dopant‐tailored matrix strategy involves rational engineering of host matrices around a preselected class of dopants by targeting matrix packing motifs that promote synergistic host‐dopant interactions upon doping. This concept, demonstrated here for dopant‐induced organic room‐temperature phosphorescence (RTP) using benzoindole‐doped carbazole matrices as a model system, provides efficient RTP materials.

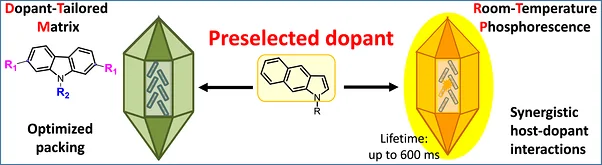

## 1 Introduction

Crystal engineering, that aims to the rational design of molecular solids with desired macroscopic properties, has emerged as a powerful strategy in materials science for building functional organic crystalline materials [1, 2]. This strategy relies on an appropriate molecular design guided by the understanding of intermolecular interactions in the context of crystal packing, with the ultimate challenge remaining the reliable prediction of crystal structures [3, 4, 5]. In particular, crystal engineering played a pivotal role in the development of organic semiconductor single‐crystals, central materials for organic photonics and electronics [6, 7]. It enabled the design of rigid π‐conjugated small molecules held together with long‐range periodic order into three‐dimensional (3D) crystal architectures by weak intermolecular interactions. These interactions determine the solid‐state packing modes of the identical molecules assemblies, which dictate electronic coupling and orbital overlap between neighboring molecules, ultimately governing the crystal electrical and optical properties [8, 9, 10, 11]. Despite these advances, a major challenge arises when a small amount of a guest molecule (dopant) is added to a host crystalline matrix to enhance its performances: how to achieve a beneficial synergistic effect between the two components that leads to improved and tuned properties, rather than detrimental effects caused by dopant‐induced disorders [12, 13]. In the present work, we address this issue by introducing the innovative and challenging crystal engineering concept of dopant‐tailored matrix (Figure 1). Unlike conventional approaches, which rely on selecting dopants with structural or size similarities to the host (Figure 1a), our alternative strategy first involves the selection of a class of dopant, and then applies crystal engineering principles to construct host matrices specifically designed to adopt targeted packing motifs that promote synergistic host‐dopant interactions upon doping (Figure 1b). We demonstrate the proof of concept of this strategy in the emerging field of long‐lived organic room‐temperature phosphorescence (RTP), and discuss its broader implications within and beyond this field.

**FIGURE 1:**
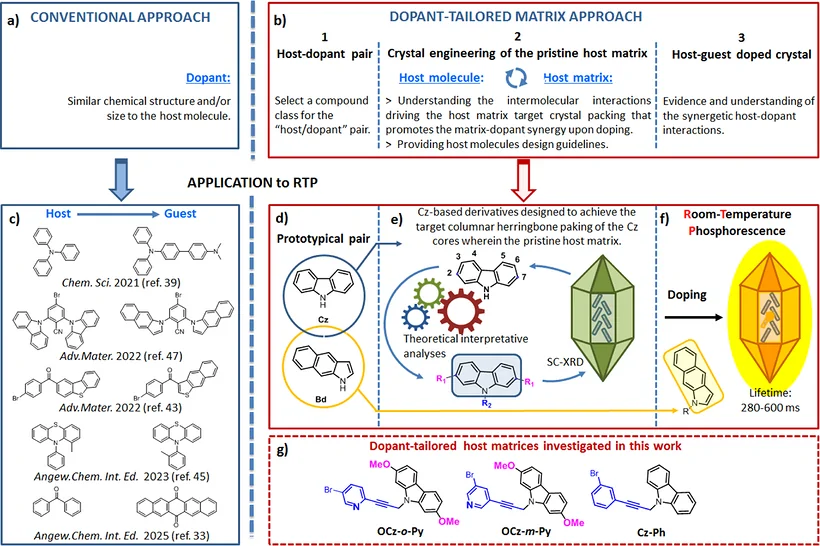
The dopant‐tailored matrix concept for the construction of host–guest doped crystals and the application to RTP materials. (a) The conventional host–guest doping approach. (b) The dopant‐tailored matrix strategy. (c) Examples of RTP host–guest systems studied in the conventional approach. (d–g) Demonstration of the dopant‐tailored matrix approach for building RTP host–guest doped crystals. Selecting the prototypical carbazole‐benzoindole (Cz‐Bd) host‐dopant pair (d), implies that the target Cz units packing motif in the pristine crystal is the columnar herringbone motif, which is achieved through the rational design of the host molecules (e). Thus, the host–guest crystal exhibits doping‐induced long‐lived RTP (f). (g) Names and structures of the dopant‐tailored host matrices.

Purely organic RTP materials exhibit persistent luminescence under ambient conditions after photoexcitation ceases, and have thus attracted increasing scientific and technological interest [14, 15, 16, 17, 18, 19, 20, 21, 22, 23], for a wide range of applications including optoelectronics [24, 25], bioimaging [26, 27, 28], X‐ray scintillators [29]. However, efficient long‐lived RTP in metal‐free organic materials remains difficult to achieve, as it requires both efficient intersystem crossing (ISC) from the lowest singlet excited state (S_1_ state) to the triplet manifold (T_n_ state), and suppression of radiationless deactivation ways from T_1_ excited state to ground state (S_0_ state) as well as quenching processes. These issues are commonly addressed through molecular design strategies that facilitate spin‐orbit coupling (SOC) and enhance the ISC, as well as by rigidifying the environment by crystallization or through host–guest strategies [30, 31, 32, 33, 34, 35]. Host–guest doped single‐crystals have therefore emerged as promising organic RTP platforms, as the crystalline host matrix can play several crucial roles. In addition to providing a rigid environment that suppresses the nonradiative deactivation pathways of guest triplet excitons, it can confine the latter and actively contribute to their generation through host–guest electronic interactions. Therefore, most strategies to construct dopant‐induced RTP host–guest crystals have focused on tuning the excited states energy levels and frontier molecular orbitals of the host and guest molecules, to achieve synergistic electronic effects [36, 37, 38, 39]. In contrast, the role of matrix crystal packing and host–guest structural synergy has remained largely unexplored [40, 41, 42], and the design of host and guest molecular structures is primarily addressed in terms of structural compatibility and similarity (Figure 1c) [43, 44, 45]. The development of rational crystal engineering strategies for RTP matrices has been hindered by both the lack of well‐defined host–dopant model systems, clearly identified target 3D matrix crystal architectures, and reliable correlation between the pristine matrix packing modes and the optical properties of the doped systems. The recent identification in commercial carbazole (Cz) of 1*H*‐benzo[*f*]indole (Bd) as an isomeric impurity, responsible for RTP in archetypal Cz‐based materials, reveals that these extensively investigated systems are intrinsically host–guest in nature [46, 47, 48, 49]. As for the mechanism of impurity‐induced RTP, although it is still under debate [49], the most recent RTP mechanisms in ‘Cz/Bd’ host/guest systems investigated in the literature both experimentally and computationally are based on the hypothesis of the diffusion in the Cz host matrix of an entity, which could be either a Cz radical anion or an exciton on the Cz [49, 50, 51]. On that aspect, we recently demonstrated that long‐lived RTP in Bd‐doped Cz‐based matrices originates from a common columnar edge‐to‐face herringbone packing motif [48]. Thus, we postulate that these findings establish the “Cz/Bd” pair as a prototypical “host–dopant” pair (Figure 1d), and identify the aforementioned packing arrangement as a key structural prerequisite for RTP in these host–guest doped crystals. Here, we hypothesize that our dopant‐tailored matrix strategy will enable to rationally design pristine carbazole‐based host matrices that adopt the herringbone crystal architecture (Figure 1e), and exhibit RTP upon doping with benzoindole derivatives (Figure 1f).

To validate this hypothesis, we designed dopant‐tailored carbazole‐based hosts (**Cz‐Ph**, **OCz‐*o*‐Py**, and **OCz‐*m*‐Py**) (Figure 1g), guided by two theoretical interpretative approaches, namely molecular electrostatic potential (MESP) and noncovalent interaction (NCI) analyses. Structural characterization demonstrates that the host matrices adopt the targeted herringbone packing of carbazole units, even in case of lateral substitution, enabled by conformational adaptability imparted by the flexible (─CH_2_─) *sp^3^* linker. Upon doping, these matrices exhibit efficient long‐lived RTP, with phosphorescence lifetimes exceeding hundreds of milliseconds, providing insight into dopant‐induced RTP mechanisms. Thus, this work establishes a general crystal engineering strategy for the rational design of host–guest systems with unprecedented precise control over solid‐state structure and optical properties, offering a versatile platform for next‐generation organic single‐crystalline semiconductors with tailored optoelectronic properties.

## 2 Results

### 2.1 Preliminaries to Molecular Design: Understanding of Intermolecular Interactions

In the context of the crystal engineering, the rational design of the new host matrices requires the understanding of the intermolecular interactions that govern and stabilize the targeted packing motif in crystals exhibiting it. In the present case, the motif is the herringbone arrangement of carbazole units, and the crystals include simple **Cz**, as well as the previously studied **Cz‐*o*‐Py** and **Cz‐*m*‐Py** (Table S1) [48].

To this end, molecular electrostatic potential (MESP) topography analysis was first employed to explore the electronic characteristics of the molecules (see Supporting Information) [52, 53, 54, 55]. This approach enables the identification of regions prone to favorable intermolecular interactions and helps rationalize molecular packing structure. This analysis aimed to identify sites of favorable intermolecular interactions and to help explain how molecules recognize and interact with each other. We first investigated the MESP of **Cz**, whose electron distribution map is shown in Figure 2a. The electron‐rich regions (red), characterized by a negative electrostatic potential of −17 kcal.mol^−1^, are mainly localized above the centers of the two six‐membered rings, corresponding to the π‐delocalized electron density. In contrast, the electron‐poor regions (blue) are located around the nitrogen atom and along the C─H bonds at the opposite edge, exhibiting positive potentials of 47 and 13 kcal.mol^−1^, respectively. Similar to the dimerization of polycyclic aromatic hydrocarbons (PAHs), two configurations could be tentatively assumed for the **Cz** dimer: the displaced staggered parallel configuration and the perpendicular (T‐shaped) structure. The most stabilizing interaction between two **Cz** molecules is expected to occur between the most positive region (N–H) of one molecule and the complementary negative region (π‐electron cloud) of another. This electrostatic complementarity favors a perpendicular (T‐shaped) arrangement (Figure 3a). In addition, π···π stacking interactions may promote a displaced parallel (cofacial) configuration. The combined contribution of these two interaction modes therefore leads to an “edge‐to‐face” (Type I) arrangement, characterized by an angle *θ* between 0° and 90°, as illustrated in Figure 3a. This MESP analysis provides a clear rationale for the single‐crystal x‐ray structure of **Cz**, which exhibits a herringbone packing motif involving the nitrogen‐containing edge. In this arrangement, molecules are interconnected through intermolecular N–H···π and C–H···π interactions, forming a herringbone angle (*θ*_herring_) of 56.72° (Figure S1a). MESP analyses were also performed on **Cz‐*o*‐Py** and **Cz‐*m*‐Py** (Figure 2b,c, respectively), using their crystallographic geometries. Both compounds display similar electrostatic features, highlighting two independent interaction sites on either side of the CH_2_ linker. First, substitution at the nitrogen atom significantly reduces the positive electrostatic potential around the N–CH_2_ linker (24 kcal.mol^−1^) compared to the N–H group in unsubstituted **Cz** (47 kcal.mol^−1^). Moreover, steric hindrance prevents the previously observed Type I “edge‐to‐face” arrangement (Figure 3b). Instead, interactions are expected between the moderately positive edge bearing the C─H bonds opposite the nitrogen atom (∼15 kcal.mol^−1^) and the negative π‐electron regions (−15 kcal.mol^−1^), resulting in a less stabilizing interaction. This leads to a Type II “edge‐to‐face” arrangement, consistent with our single‐crystal X‐ray diffraction data (Figure S2) and with reports in the literature on π‐conjugated materials [48]. The second key feature concerns the pyridine ring. The pyridinic nitrogen exhibits a strong negative electrostatic potential (∼ −30 kcal/mol), while the hydrogen atom located opposite to it shows a pronounced positive potential (24 kcal/mol). This electrostatic complementarity drives here directional intermolecular N···H–C hydrogen bonding between pyridine rings, leading to the formation of supramolecular chains along the stacking direction (Figure S2).

**FIGURE 2:**
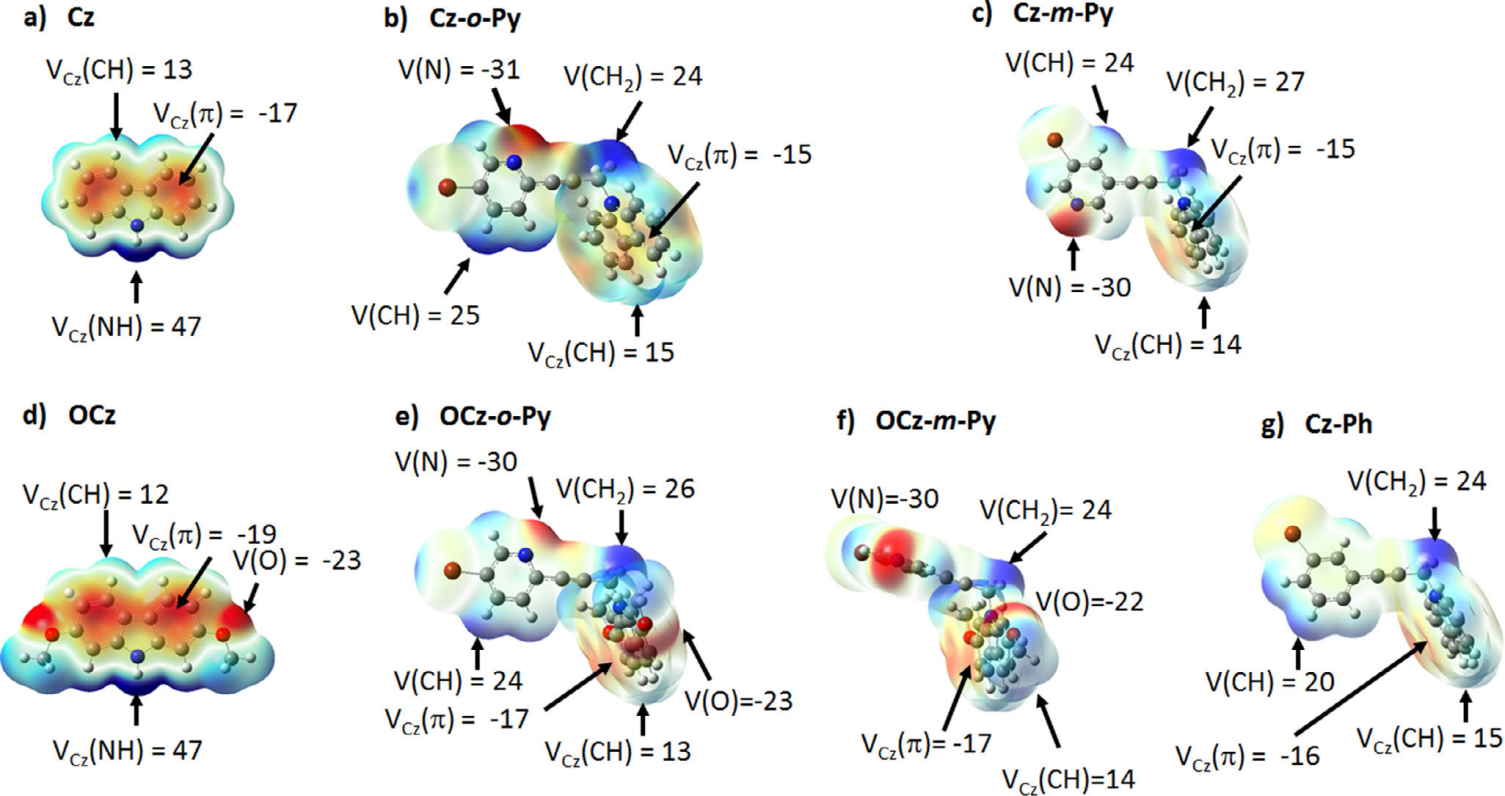
MESP isosurfaces on the 0.001 au electron density isosurfaces. The potential values of some relevant points are given in kcal/mol. (a) **Cz**, (b) **Cz‐*o*‐Py**, (c) **Cz‐*m*‐Py**, (d) **OCz**, (e) **OCz‐*o*‐Py**, (f) **OCz‐*m*‐Py**, and (g) **Cz‐Ph**.

**FIGURE 3:**
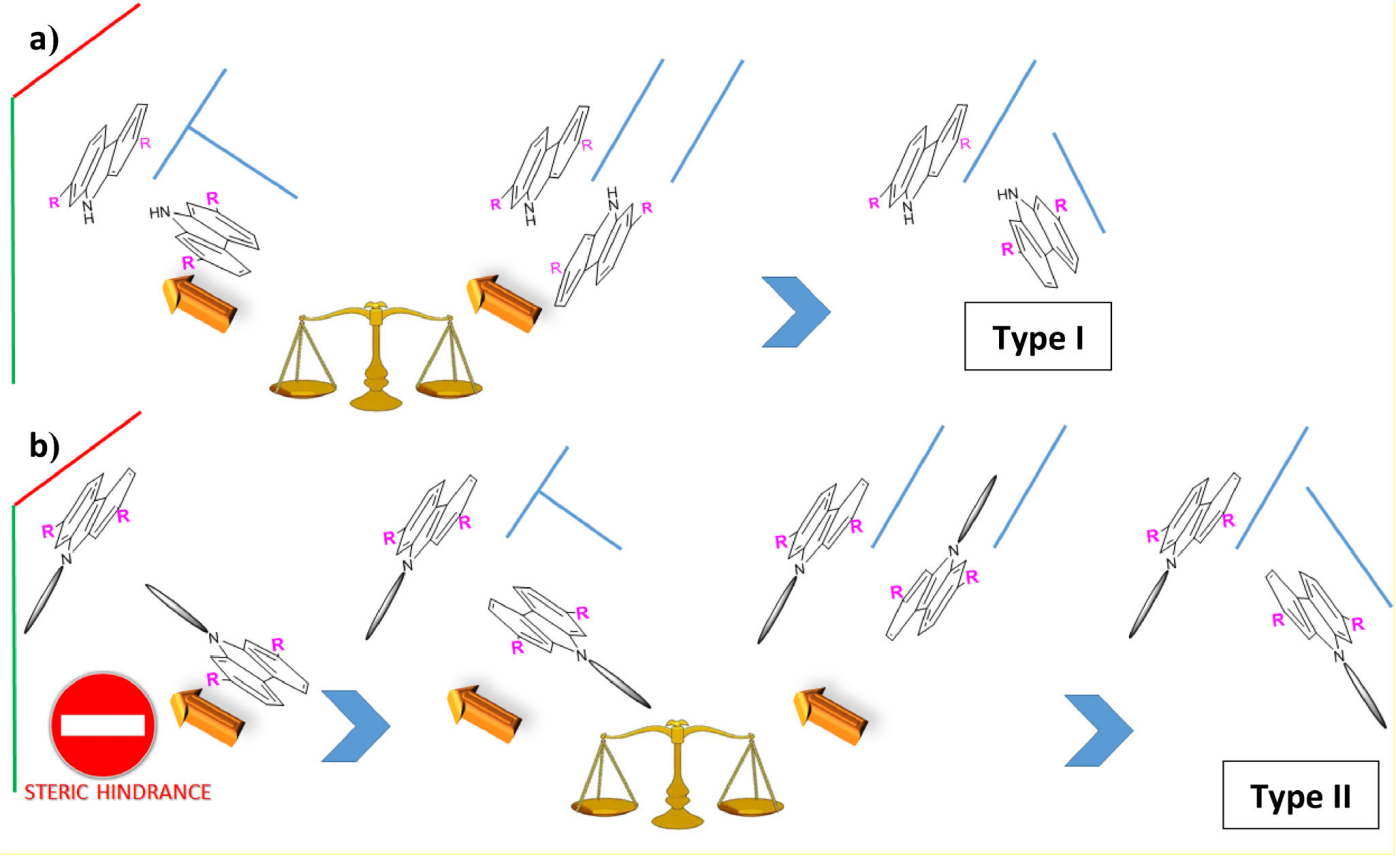
Sketch of the two types of edge‐to‐face herringbone packing formation. Balance between electrostatic forces as deduced from MESP analysis and π^…^π stacking in (a) carbazole and carbazole derivatives substituted at 2,7‐positions, (b) carbazole derivatives substituted at the N‐atom. R = H, OMe.

For a deeper understanding, non‐covalent interaction (NCI) analysis was additionally performed to visualize the intermolecular interactions [56, 57, 58]. Figure 4a shows the NCI representation of a **Cz** trimer extracted from the crystal structure. It highlights the presence of three different intermolecular regions (shown in green) responsible of the attractive interactions between the carbazole cores. The two regions labelled as Reg.1 and Reg.2 correspond to the “edge‐to‐face” Type I and II, respectively, while Reg.3 represents the intermolecular interaction between two adjacent parallel carbazole molecules. In the case of the **Cz‐*o*‐Py** and **Cz‐*m*‐Py** crystals, the NCI analysis (Figure 4b,c, respectively) reveals four intermolecular regions. However, regions Reg.1 and Reg.2 clearly correspond here to the Type II interaction, while the third region (Reg.3) is again associated with the intermolecular interaction between two adjacent parallel carbazole cores. Finally, the fourth region (Reg.4) reveals the hydrogen bond between adjacent pyridine units.

**FIGURE 4:**
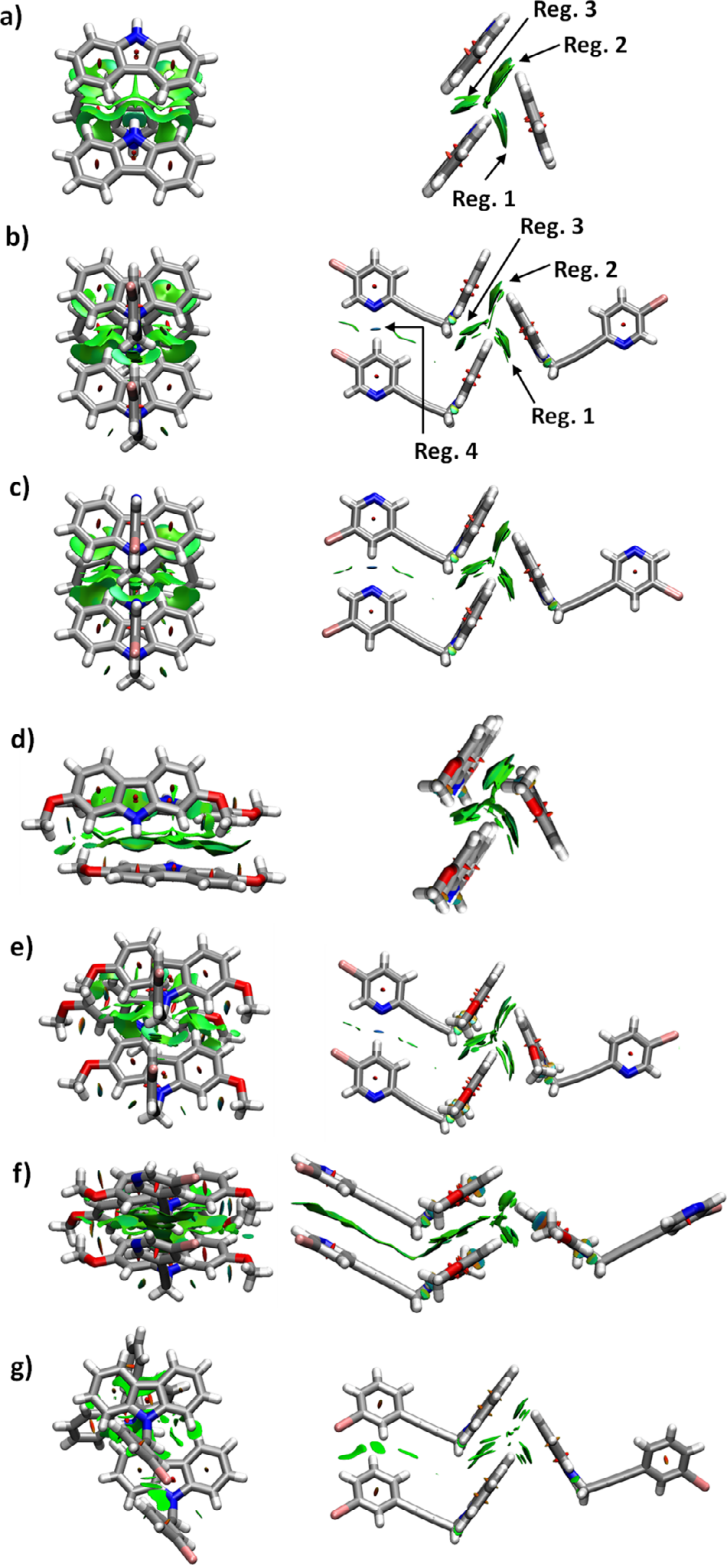
NCI analysis of (a) **Cz**, (b) **Cz*‐o*‐Py**, (c) **Cz*‐m*‐Py**, (d) **OCz,** (e) **OCz*‐o*‐Py,** (f) **OCz*‐m*‐Py,** and (g) **Cz‐Ph**. Color signification are the classical ones as proposed by the NCI developers (green slightly attractive interactions, blue strong attractive interaction, red repulsion).

All these results allow us to highlight several important trends that can guide the further design of molecular crystal architectures exhibiting the herringbone motif. First, it has been shown that two types of “edge‐to‐face” (Type I and Type II) can be obtained depending on the substitution of the N atom of the carbazole core. Second, the existence of the intermolecular interaction between two adjacent parallel carbazole cores stabilizes the herringbone motif along the stacking axis. Third, regardless of the intermolecular interaction involved, only the two axial CH groups (2,7‐positions) can be substituted without disrupting the herringbone structure, as they are not involved in this motif, unlike the other CH groups at the edges (e.g., 3, 4, 5, 6‐positions).

### 2.2 Molecular Design and Synthesis of Target Hosts

In this context, molecules **OCz‐*o*‐Py, OCz*‐m*‐Py**, and **Cz‐Ph** were designed (Figure 1g). They all have a σ‐conjugated skeleton in which the methylene ‐CH_2_‐ linker (*sp^3^)* is expected to act as a rotula that allows for conformational adaptability. Two cases are considered regarding the substructures on either side of the ‐CH_2_‐ group. The goal is to tune the aforementioned intermolecular interactions that influence the substructures self‐assembly and ultimately stabilize the herringbone stacking of the carbazole units in the crystal. In one case, the pyridine motif remains while the carbazole ring undergoes functionalization. Based on the aforementioned results, we speculate that substituting the two axial terminal hydrogen atoms (in the 2,7 positions) of the carbazole cores will preserve the edge‐to‐face interactions, and thus will result in less disruption to the herringbone packing motif in the matrix. This results in molecules **OCz‐*o*‐Py** and **OCz‐*m*‐Py**, daughter derivatives of parent molecules **Cz‐*o*‐Py** and **Cz‐*m*‐Py**, respectively. Alternatively, the carbazole ring is retained undecorated, and the variation involves the moiety responsible for hydrogen bonding. Thus, the pyridine ring is replaced with a benzene ring to suppress this contribution. The result is compound **Cz‐Ph**, a daughter molecule of **Cz‐*m*‐Py**.

The synthesis of the target molecules is adapted from our previous works by replacing (i) the carbazole core with the 2,7‐dimethoxy‐9H‐carbazole (**OCz**) core for **OCz‐*o*‐Py** and **OCz‐*m*‐Py**, and (ii) the 3‐bromo‐2‐iodopyridine with the 1‐bromo‐4‐iodobenzene for **Cz‐Ph** (see Supporting Information). The compounds were fully characterized by ^1^H‐ and ^13^C‐NMR spectroscopy and mass analysis (see Supporting Information). Single crystals were grown by slow evaporation from a mixture of *n*‐hexane and methylene chloride at room temperature and analyzed by single‐crystal x‐ray diffraction (SC‐XRD) measurements (see Supporting Information).

### 2.3 Single Crystal X‐Ray Diffraction, MESP, and NCI Analyses of Pristine Host Matrices

In a first stage, the crystal structures of **OCz** and pure **Cz** were compared to study how the addition of two bulky methoxy groups at the 2,7‐positions affects the packing of the carbazole rings. The crystallographic analysis of **OCz** and **Cz** reveals strong similarities (Figure S1). Both compounds belong to an orthorhombic space group, and adopt a typical two‐dimensional (2D) layered‐herringbone structure with an “edge‐to‐face” arrangement along the long molecular axis with similar herringbone angles (*θ*_herring_) of 59.17 and 56.72° for **OCz** and **Cz**, respectively. In addition, the interplanar distances (*d*_inter_) between aromatic rings within a given stack are also quite identical for **OCz** and **Cz**, 2.76 and 2.70 Å, respectively. The only notable difference resulting from the lateral substitution of the carbazole core with bulky methoxy groups is an increase in the lattice parameter along the long molecular axis, which is 25.76 Å for **OCz** and 19.05 Å for **Cz**. The MESP analysis reveals a potential distribution map (Figure 2d) closely resembling that of **Cz**, with the exception of an additional electron‐rich region (−23 kcal·mol^−^^1^) located on the oxygen atom. The formation of the herringbone arrangement can therefore be rationalized by the scheme shown in Figure 3a. On Figure 4d, we can visualize the NCI representation of a **OCz** trimer extracted from the crystal structure. Similar to the **Cz** case, we clearly see that the substituted groups located at 2,7‐position do not contribute to the non‐covalent interactions involved in the herringbone structure, and thus do not affect strongly the herringbone packing of **OCz**.

We then studied the crystal structure of the **OCz‐*o*‐Py** target molecule (Figures 5a,b, and S3a), and compared it to those of its parent molecule (**Cz‐*o*‐Py**) and of **OCz**. Figure S3a reveals that the **OCz‐*o*‐Py** molecules adopt in the crystal a nearly perpendicular conformation between the carbazole long axis and the pyridine ring, in the very same way as the **Cz‐*o*‐Py** molecules (Figure S3b). On the other hand, Figures 5a,b show that the crystal structure of **OCz‐*o*‐Py** combines the main features of its parent **Cz‐o‐Py** and of **OCz**. First, the substituted carbazole cores of **OCz‐*o*‐Py** molecules and carbazole units of **Cz‐*o*‐Py** molecules pack into columns along the *b* axis forming a herringbone‐like structure with quite similar edge‐to‐face angles (*θ*_herring_ = 54.28 and 54.68°, respectively) (Figures 5a and S2a, respectively). Second, the **OCz‐*o*‐Py** dimethoxycarbazole units stack along the *b* axis with an interplanar distance *d*_inter_ (2.72 Å), which is nearly identical to the distance in **Cz‐*o*‐Py** (2.73 Å). As well, the pyridine rings in the **OCz‐*o*‐Py** crystal self‐assemble by hydrogen bonding along the *b* axis (N….H‐C = 2.28 Å) as observed for **Cz‐*o*‐Py** crystal. Most importantly, as shown by the comparison of Figures 5b and S2b, the substituted carbazole‐based columns of the **OCz‐*o*‐Py** crystal self‐assemble in such a way that (i) in the *a‐c* plane, adjacent pairs of edge‐to‐face carbazole units adopt along the long molecular axis an organization quite similar to that of the **OCz** crystal, and (ii) in the perpendicular *b* direction, the planes containing the pyridine rings and the ethynyl linkage adopt a face‐to‐face organization quite similar to that of the **Cz‐*o*‐Py** crystal with an interplane distance (*d*_pyr‐pyr_) of 3.20 Å vs 3.33 Å. In addition, comparison of the MESP representations of **OCz‐*o*‐Py** and its parent compound **Cz‐*o*‐Py** (Figure 2e,b, respectively) indicates very similar electrostatic potential distributions, except for the presence of an additional negative potential (−17 kcal·mol^−^^1^) localized on the oxygen atom of the methoxy substituent. Furthermore, the NCI analysis of **OCz‐*o*‐Py** crystal (Figure 4e) reveals very similar noncovalent interactions as for **Cz‐*o*‐Py** (Figure 4b), both for carbazole cores and pyridine cycles. Therefore, as expected, the functionalization of the carbon at 2,7‐positions of the carbazole core does not affect the supramolecular arrangement of the molecules along the stacking axis. Overall, the MESP and NCI analyses reveal that the nature of the intermolecular interactions within the molecular crystals remains essentially unchanged when transitioning from the non‐methoxylated parent compound (**Cz‐*o*‐Py**) to the methoxylated daughter molecule (**OCz‐*o*‐Py**).

**FIGURE 5:**
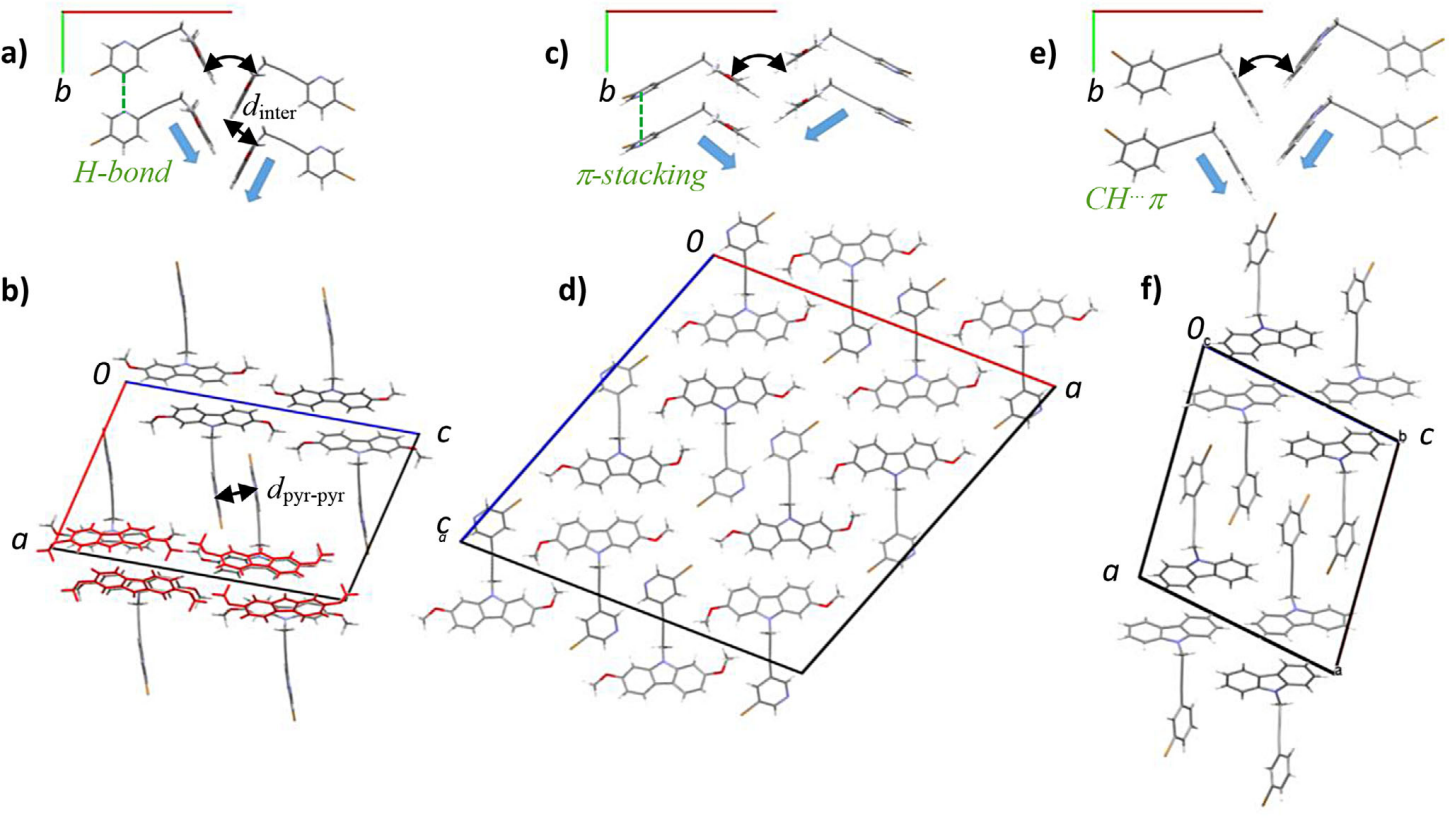
Crystal structures of OCz‐*o*‐Py, OCz‐*m*‐Py, and Cz‐Ph. (a) Molecular stacking of **OCz‐*o*‐Py** along the *b*‐axis. (b) Arrays of **OCz‐*o*‐Py** in the *a‐c* plane. Superimposed in red, arrays of **OCz**. (c) Molecular stacking of **OCz‐*m*‐Py** along the *b*‐axis. (d) Arrays of **OCz‐*m*‐Py** in the *a‐c* plane, (e) Molecular stacking of **Cz‐Ph** along the *b*‐axis. (f) Arrays of **Cz‐Ph** in the *a‐c* plane. The blue arrows represent the molecular dipole moments. In green, the kind of intermolecular interaction involved in the non‐carbazole‐based subunit.

As for the **OCz‐*m*‐Py** crystal, the molecules adopt a drastically different conformation than the **Cz‐*m*‐Py** parent molecules and **OCz‐*o*‐Py** molecules (Figures S4 and S5, respectively). As shown in Figure S4, the twist angles of the pyridine ring around the triple bond (*θ_1_ =* 38.46°) and between the carbazole and methylene spacer (*θ_2_* = 32.08°) in **OCz‐*m*‐Py** are indeed much smaller than their counterparts in **Cz‐*m*‐Py** (57.26° and 51.13°, respectively). Despite these molecular conformational changes, the dimethoxycarbazole units stack perfectly in a herringbone‐like pattern (Figure 5c), which is similar to that observed for the unsubstituted **Cz‐*m*‐Py** carbazole one (Figure S2c). The edge‐to‐face angle *θ*_herring_ is 65.37° and the interplanar distance is 3.44 Å, compared to 54.68° and 2.75 Å, respectively, for **Cz‐*m*‐Py**. Furthermore, the dimethoxycarbazole‐based herringbone columns are shown to be packed in arrays similar to those of **Cz‐*m*‐Py** (Figures 5d and S2d). However, the **OCz‐*m*‐Py** conformation precludes self‐assembly of the pyridine rings by hydrogen bonding along the stacking axis, but rather favors their cofacial π‐packing with an interplanar distance of 3.7 Å. The results are well supported by the MESP and NCI representations (Figures 2f and 4f, respectively).

In the **Cz‐Ph** crystal, molecules adopt a conformation where the twist angle between the carbazole and methylene spacer (*θ_2_* = 40.59°) does not differ significantly from that in **Cz‐*m*‐Py** (Figure S6). Consequently, compared to **Cz‐*m*‐Py** (Figure S2c), the packing into columns along the *b* axis forming a herringbone‐like structure with quite similar edge‐to‐face angles (*θ*_herring_ = 68.12°) and interplanar distances (*d*_inter_ = 3.12 Å) between aromatic rings within a given stack is preserved (Figure 5e). However, the twist angle (*θ_1_ =* 72.81°) is larger than in **Cz‐*m*‐Py**, reflecting a difference compared to the perpendicular conformation between the carbazole long axis and the benzene ring (Figure S6). Additionally, the loss of hydrogen bonds due to replacing pyridine rings with benzene rings is balanced by a herringbone stacking of benzene rings along the *b* axis (*θ*_herring_ = 58.95°), as shown in Figure S7. The NCI analysis supports these results since the replacement of the bromo‐pyridine by a bromo‐phenyl does not affect the interactions between the Cz units in the herringbone pattern (Figure 4g). Finally, Figure 5f shows that **Cz‐Ph** molecules form in the crystal a supramolecular 2D network of carbazole unit columns, which is quite similar to that observed for **Cz‐*m*‐Py**. (Figure S2d).

### 2.4 Photophysical Properties

The photoluminescence (PL) properties of **OCz‐*o*‐Py** and **OCz‐*m*‐Py** in EtOH/MeOH (80:20) solutions (10^−5^ M) at 77K were investigated. When excited with a 365 nm UV lamp, both frozen solutions display a long blue afterglow visible to the naked eye after the excitation was turned off (Figure 6a). When excited at 310 nm, these **OCz‐*o*‐Py** and **OCz‐*m*‐Py** frozen solutions have similar steady state PL spectra, which are characterized by an intense band with well‐defined peaks at 340 and 357 nm and a broader weaker signal centered at 450 nm (Figure 6a). As shown in Figure 6a in the case of **OCz‐*o*‐Py**, only this low‐energy‐band is present upon time‐gating the measurement. Thus, from these spectra, the short‐ and long‐wavelength transitions are attributed to fluorescence and phosphorescence, respectively. From the onset on the luminescent spectra, the corresponding energies of the lowest energy molecular singlet (S_1mol_) and triplet (T_1mol_) excited states are estimated at 3.75 and 3.13 eV, respectively. Since the PL properties of **OCz‐*o*‐Py** and **OCz‐*m*‐Py** in frozen solutions are similar to those of their parents **Cz‐*o*‐Py** and **Cz‐*m*‐Py**, respectively, it can be deduced that the methoxy substituents do not induce any significant change, at the molecular level, of the electronic properties of the daughter molecules. This is further confirmed by quantum chemical calculations of excited state energies (Figure S8). The absorption energy for the S_0_‐S_1_ transition of all the molecules is computed at around 4 eV (∼310 nm) while the fluorescence is estimated around 3.5 eV (∼350 nm) and the phosphorescence at 3.0 eV (∼410 nm). These transition energies are quantitatively in good agreement with the experimental results. This also indicates that thermally activated delayed fluorescence is not favored since the ΔE_S‐T_ is as great as 500 meV. Compared to the frozen solutions, both the **OCz‐*o*‐Py** and **OCz‐*m*‐Py** host matrices exhibit a bright green afterglow in their pure crystalline form at 77K after the UV lamp was turned off (Figure 6b). This emission is reflected on the PL spectra by a broad band at 550 nm, which dominates the delayed phosphorescence spectrum, as shown in Figure 6b for **OCz‐*o*‐Py**. Accordingly, time resolved photoluminescent measurements were performed and confirmed the long‐lived nature (lifetime ∼690 ms) of the excited state involved for this transition (Figure S9). This new red‐shifted phosphorescence band is clearly distinct from the one previously observed at 450 nm in dilute frozen solution, and ascribed to the molecular triplet excited state. Therefore, we can infer that this lower energy matrix triplet excited state (T_1mat_) is certainly the results of molecular aggregation in a dimer or higher aggregated state feature, as already reported for dimer or excimer species based on carbazole [59]. The hypothesis that the lowering of this transition originates from multichromophoric species is supported by the absence of species that are computed by quantum chemistry at or lower wavelength than 550 nm (in energy ∼2.2 eV) for isolated molecules. On the other hand, at RT, the **OCz‐*o*‐Py** and **OCz‐*m*‐Py** matrices exhibited only weak emission under UV excitation and no afterglow was visible to the naked eye when the UV lamp was turned off. However, steady‐state PL spectra display two broad emission peaks: one centered at 375 nm with a lifetime of 40.8 ns, demonstrating its fluorescence nature, and another one at 550 nm with even weaker intensity and a lifetime of 1.8 ms, ascribed as at 77K to phosphorescence (Figure S10). This suggests that the T_1mat_ triplet excited state observed at 77 K can also form at RT, but the increase in temperature considerably shortens its lifetime and reduces its intensity.

**FIGURE 6:**
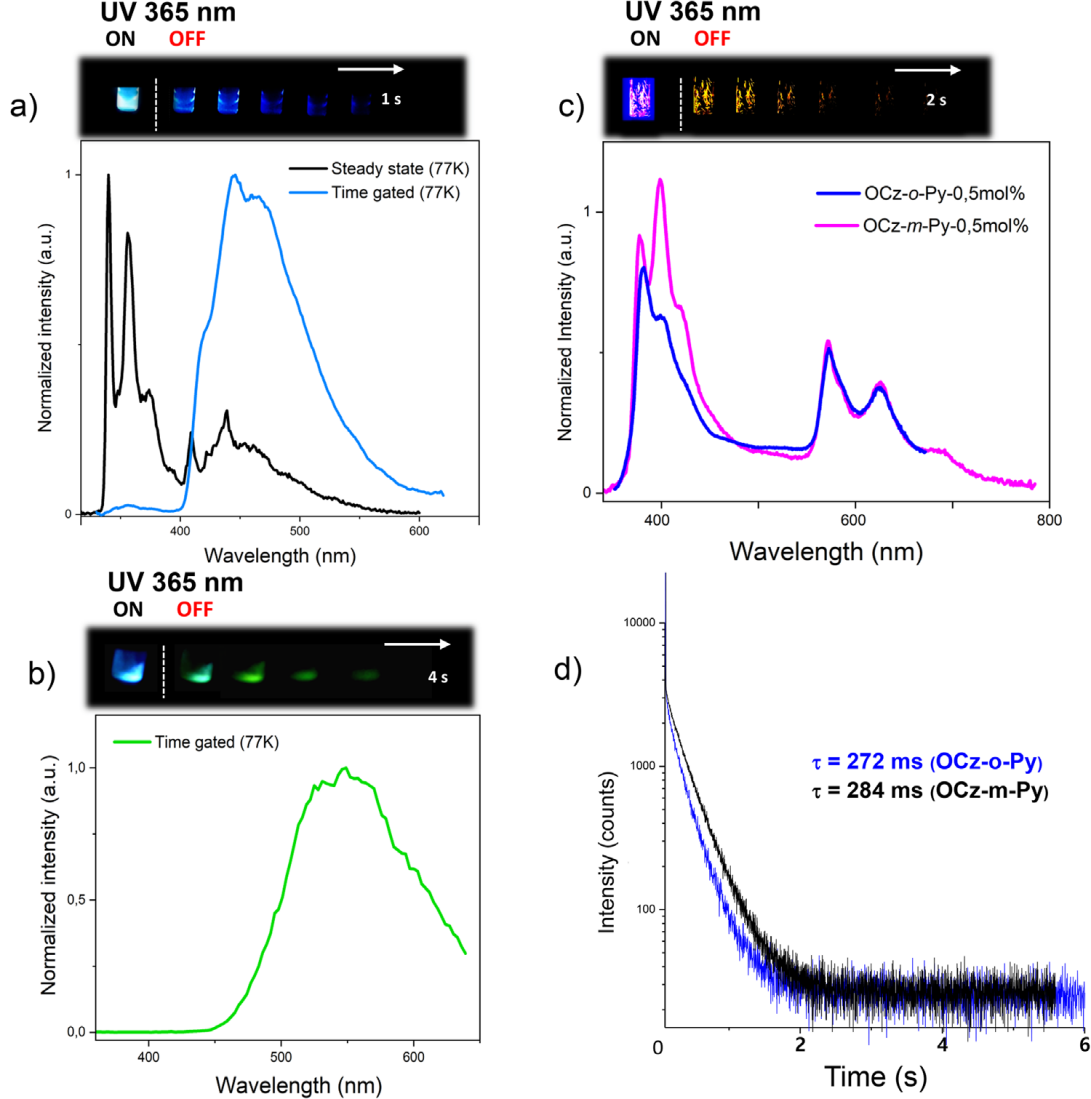
Photoluminescence properties of pristine and doped matrices. (a) Photographs of the isolated molecules **OCz‐*o*‐Py** (**OCz‐*m*‐Py)** in diluted frozen solution at 77K (10^−5 ^M, EtOH/MeOH 80:20) under a 365 nm UV lamp ‘On‘ and ‘Off‘, and steady‐state photoluminescence (black line) and phosphorescence (blue line) spectra of **OCz‐*o*‐Py** in frozen diluted solution at 77K (10^−5 ^M, EtOH/MeOH 80:20). (b) Photographs of **OCz‐*o*‐Py** (**OCz‐*m*‐Py)** crystal at 77K under 340 nm excitation, and phosphorescence spectra of **OCz‐*o*‐Py** (**OCz‐*m*‐Py)** crystal at 77K under 340 nm excitation. (c) Photographs of doped **OCz‐*o*‐Py** (**OCz‐*m*‐Py)** crystals at RT under a 365 nm UV lamp ‘On‘ and ‘Off‘, and steady‐state photoluminescence of doped **OCz‐*o*‐Py** (blue curve) and **OCz‐*m*‐Py** (red curve) doped under 340 nm excitation at RT. (d) Photoluminescence decay curves at RT of doped **OCz‐*o*‐Py** (blue curve) and **OCz‐*m*‐Py** (black curve) crystals.

We next examined the photoluminescent properties of the doped **OCz‐*o*‐Py** and **OCz‐*m*‐Py** crystals, in which we incorporated the Bd‐based dopant **Bd‐*o*‐Py** and **Bd‐*m*‐Py**, respectively (Table S1). The latter are isomers of the parent molecules **Cz‐*o*‐Py** and **Cz‐*o*‐Py,** respectively. Compared to the pristine matrices, the doped systems revealed a new photophysical behavior mainly characterized by a long orange afterglow upon switching off the 365 nm UV‐lamp (Figure 6c). As shown on Figure 6c, the steady‐state photoluminescence spectra of these two host–guest systems exhibit a dual emission comprising a short wavelength band with peaks at 381, 403, and 424 nm, while another structured signal typical of Bd‐doped carbazole‐based systems appeared at longer wavelength, with three peaks located at 573, 627, and 688 nm. The recorded lifetimes of the short wavelength emission belong to the fluorescence range with values of 1.7 and 4.0 ns. In contrast, the phosphorescence nature of the long wavelength emission is demonstrated from the time resolved photoluminescent measurements for which lifetimes of 272 and 284 ms are detected for the doped **OCz‐*o*‐Py** and **OCz‐*m*‐Py** crystals, respectively (Figure 6d). This emission is ascribed to the Bd‐based dopant RTP, whose PL spectra displays identical emission signature than in glassy solution or doped PMMA film at 77K [48]. This emission from the benzoindole dopant triplet state is further supported by post‐Hartree–Fock CC2 calculations predicting triplet state emission at 2.2 eV (∼560 nm), as shown in Figure S8. Finally, the main distance between component peaks is on average 0.18 eV, ∼1450 cm^−1^, a value in the range of molecular vibration modes, particularly C‐N and N‐H modes of pyrrole ring of the benzoindole unit [60], supporting the hypothesis of a vibronic coupling.

Additionally, the photoluminescence properties of a **Cz‐Ph** matrix synthesized from commercial carbazole, and thus intrinsically doped with its isomeric “impurity,” have been investigated (Figure S11). The steady‐state spectrum shows the typical dual emission consisting of short‐wavelength peaks and a structured signal at longer wavelengths. The former emission is attributed to fluorescence, with lifetimes of ∼1.3 ns, while the latter, with the characteristic vibronic progression and lifetimes of ∼611 ms is attributed to the phosphorescence of the **Cz‐Ph** isomeric benzoindole‐based dopant.

## 3 Discussion

The edge‐to‐face herringbone packing motif is a common pattern in organic semiconductors and was recently identified as a critical structural arrangement enabling dopant‐induced RTP in carbazole‐based systems [48], which are representative of organic RTP materials. Here, we report at first a crystal engineering strategy aimed at stabilizing this packing mode through an in‐depth understanding of the intermolecular interactions governing this crystalline organization. As evidenced in previous literature on several RTP host‐guest systems [39, 40, 46, 48], and as we recently demonstrated experimentally and by computational modeling on parents of our Cz/Bd systems [48, 51], we can reasonably assume that the Bd‐based dopant does not modify the packing of the host, at least at the concentration (0.5%) considered in this work. Combining experimental single crystal x‐ray diffraction analysis and multiscale theoretical interpretative approaches performed on simple carbazole (**Cz**) and previously reported carbazole‐based parent matrices (**Cz‐*o*‐Py, Cz‐*m*‐Py**), we elucidate the interplay of intermolecular interactions that control this pattern as well as the role played by the carbazole core substitution. In all cases, aromatic edge‐to‐face interactions between carbazole cores remain the dominant driving force. Based on these insights, we designed new daughter derivatives with a σ‐conjugated backbone (**Cz‐Ph**) and judicious carbazole core substitutions (**OCz‐*o*‐Py, OCz‐*m*‐Py**) to preserve the interactions behind the targeted herringbone motif. Comparative structural analyses of parent and daughter derivatives reveal that the central methylene (─CH_2_─) *sp^3^* linker provides conformational flexibility, while allowing various supramolecular interactions, such as hydrogen bonding (**OCz‐*o*‐Py**), π‐stacking (**OCz‐*m*‐Py**), and CH^…^π contacts (**Cz‐Ph**), to coexist on one side of the linker without disrupting the carbazole herringbone motif on the other (Figure 5a). These results demonstrate that the subunits on either side of the methylene group self‐organize within the crystal in a supramolecular way, creating cooperative effects, which synergistically stabilize the target crystal structure. Overall, our successful methodology could be extended to other (hetero‐)acenes, and our findings highlight the relevance of σ‐conjugated architectures as a powerful crystal engineering tool for controlling packing motifs in organic semiconductor single‐crystals.

Beyond structural control, we show that the dimensionality of the herringbone network can be deliberately tuned, with possible consequences on charge‐transport properties. The optical and electronic properties of small‐molecule organic semiconductors strongly rely on their molecular packing, which determines the degree of neighboring molecular orbital overlap (i.e., charge transfer integrals) and defines charge transport pathways [9, 10]. While conventional layered edge‐to‐face herringbone arrangements promote predominantly two‐dimensional charge transport [9], as exemplified for **Cz** and **OCz** (Figure S12a), the columnar herringbone organization observed in our σ‐conjugated matrices (e.g. **OCz‐*o*‐Py** and **OCz‐*m*‐Py**) and previously reported π‐conjugated carbazole‐based materials, suppresses intercolumnar electronic coupling, confining charge transport to a strongly anisotropic one‐dimensional pathway along the carbazole‐based column axis (Figure S12b). Since carbazole is a widely used building block in optoelectronic materials, such control over transport dimensionality should provide new opportunities for designing organic semiconductors with directional charge‐transport channels.

Ultimately, we successfully validate the tailored‐dopant matrix approach for achieving long‐lived dopant‐induced RTP. The carbazole‐based matrices engineered to adopt a columnar herringbone arrangement exhibit RTP with lifetimes ranging from 250 to 610 ms upon doping with Bd‐based derivatives. Table S6 shows that this performance, particularly in terms of lifetime, ranks among the highest. Our findings raise two key questions: can we gain a better understanding of still under debate mechanisms governing RTP in host‐guest doped systems, and what role does the molecular structure play? Although a charge trap/detrap model involving charge‐separated states between Bd and Cz cores and diffusion of Cz radical anion has been proposed for related systems [46, 50], several arguments are not in favor of such a mechanism. First, carbazole is intrinsically a hole‐transporting material, favoring stable Cz radical cation hopping, rather than an electron transporting materials [61]. Second, the methoxy substitution of carbazole in **OCz‐*o*‐Py** and **OCz‐*m*‐Py** is expected to destabilize the radical anions. Third, comparable RTP lifetimes are observed across systems with markedly different transport dimensionalities, including both simple Cz or σ‐ and π‐conjugated carbazole‐based systems doped with Bd [46, 48]. Together, these results indicate that RTP is not governed by Cz anion diffusion through the Cz matrix. Instead, they support a plausible alternative mechanism in which the crystalline carbazole‐based matrix behaves as a rigid solid solution, providing a confined and dilute environment that stabilizes Bd triplet excited states, restricts nonradiative deactivation, and prevents aggregation‐induced quenching. However, unlike amorphous matrices or solid solutions, the crystalline carbazole‐based host is not passive, but could actively contributes to triggering the phosphorescence of the dopant through synergistic electronic and structural effects. The RT photoluminescence properties of the matrix could enable energy transfers from its singlet and triplet excited states to those of the dopant as strongly supported by quantum chemical calculations on the Cz/Bd system and some parents of our systems [50, 51]. Thus, excitons generated within the matrix could either transfer directly to nearby Bd units or diffuse along the herringbone columns prior to transfer, a process particularly efficient in single‐crystal matrices, because of the long‐range order and low defect density.

Finally, since our results and the literature demonstrate that the RTP features remain nearly similar regardless type of conjugation and substitution of the matrix or dopant [46, 47, 48], we can therefore infer that the substituents primarily serve a critical structural role by stabilizing the herringbone packing of the matrix rather than modulating the triplet localized excited (^3^LE) state of the dopant. This opens avenues for functionalizing the carbazole core to introduce additional properties while preserving the herringbone motif, paving the way towards new multifunctional RTP host‐guest doped systems.

## 4 Conclusion

In summary, we established a dopant‐tailored matrix crystal engineering strategy for the construction of host–guest doped single crystals, in which a specifically designed matrix organization is targeted for promoting synergistic structural interactions with the guest dopant. As a proof of concept, this approach is applied to dopant‐induced organic long‐lived room‐temperature phosphorescence. In the context of the prototypical “carbazole–benzoindole” host–dopant system, we demonstrate the rational design of carbazole‐based derivatives capable of forming pristine host matrices with a targeted columnar herringbone packing of carbazole cores. Guided by multiscale theoretical interpretative analyses of single‐crystal XRD data, we elucidate the key intermolecular interactions governing the stabilization of this edge‐to‐face motif. Furthermore, we highlight the critical role of the σ‐conjugated molecular architecture as a versatile platform, which provides conformational adaptability and can accommodate additional supramolecular interactions without disrupting the carbazole herringbone packing. Importantly, this approach allows deliberate control over the dimensionality of the herringbone network, from layered to columnar organizations, offering new opportunities to tailor charge‐transport pathways in carbazole‐based materials. Beyond the structural control, we validate the effectiveness of these engineered matrices as efficient hosts for long‐lived RTP upon doping with benzoindole derivatives, and propose an alternative RTP mechanism in these systems. The proposed strategy, which is centered on the Cz/Bd model system here, could be extended to other recently discovered host‐dopant combinations, including for example the dibenzothiophene (DBT)‐naphtho[2,3‐b]thiophene (NT) and dibenzofuran (DBF)‐naphto[2,3‐b]furan (NF) host‐dopant pair [43, 49]. Finally, this work could establish dopant‐tailored crystal engineering as a new paradigm for designing functional doped organic semiconductor crystals with tailored optoelectronic properties.

## Author Contributions

**Catherine Demangeat**: investigation, writing – original draft, conceptualization, methodology, validation, writing – review and editing. **Raphael Rullan**: investigation, validation. **Yipeng Tang**: investigation, methodology, writing – original draft, validation. **Bin Hu**: investigation, methodology, writing – original draft, validation, funding acquisition, supervision. **Anthony D'aléo**: investigation, funding acquisition, conceptualization, validation, writing – original draft. **Tangui Le Bahers**: investigation, conceptualization, writing – original draft, supervision, validation. **Mohammad Esmail Alikhani**: conceptualization, writing – original draft, validation, methodology. **André‐Jean Attias**: writing – original draft, conceptualization, funding acquisition, methodology, validation, writing – review and editing, project administration, supervision.

## Conflicts of Interest

The authors declare no conflicts of interest.

## Supporting information




**Supporting File**: anie73823‐sup‐0001‐SuppMat.docx.

## Data Availability

The data that supports the findings of this study are available in the supplementary material of this article
