# Supplementary material for: Dopant‐Tailored Matrices: A Crystal Engineering Strategy for Organic Room‐Temperature Phosphorescent Host–Guest Systems and Beyond
Source: Angew Chem Int Ed Engl. 2026 Aug 12;65(41):e8252460. doi: 10.1002/anie.8252460 (PMC13638094; doi:10.1002/anie.8252460)
Supplement: Supplementary file 1 [file ANIE-65-e8252460-s001.docx]

**SUPPORTING INFORMATION**

**Dopant-Tailored Matrices: A Crystal Engineering Strategy for Organic Room-Temperature Phosphorescent Host–Guest Systems and Beyond.**

**Authors:**

Catherine Demangeat,^1,2^ Raphael Rullan,^3^ Yipeng Tang,^4^ Bin Hu,^4^ Anthony D’Aléo,^5^ Tangui Le Bahers,^3^ Mohammad Esmail Alikhani,^6^ André-Jean Attias*^1,6^

**Affiliations:**

^1^ Dr. C. Demangeat, Prof. A.-J. Attias

Building Blocks for FUture Electronics Laboratory, IRL 2002, CNRS-Sorbonne Université-Yonsei University, Yonsei University, 50 Yonsei-ro, Seodaemun-gu, Seoul, 03722, Republic of Korea.

^2^ Dr. C. Demangeat

Department of Engineering Science, The University of Electro-Communications, 1-5-1 Chofugaoka, Chofushi, 1828585, Tokyo, Japan.

^3^ R. Rullan, Prof. T. Le Bahers

ENS de Lyon, CNRS, LCH, UMR 5182, 69342, Lyon cedex 07, France.

^4^ Dr. Y. Tang, Prof. B. Hu

State Key Laboratory of Luminescent Materials and Devices, South China University of Technology, Guangzhou 510640, P.R.China.

^5^ Dr. A. D’Aléo

Institut de Physique et Chimie des Matériaux de Strasbourg (IPCMS), UMR 7504, CNRS-Université de Strasbourg, 67034 Strasbourg Cedex 2, France.

^6^ Prof. M. E. Alikhani, Prof. A.-J. Attias

Sorbonne Université, CNRS, MONARIS - UMR 8233, 75005 Paris, France

Email: andre-jean.attias@sorbonne-universite.fr

**Abstract:**

Host–guest doping of molecular crystals is a powerful strategy to tune optoelectronic properties, yet achieving precise host–dopant compatibility and beneficial synergy without introducing detrimental effects caused by dopant-induced disorders remains challenging. Here, a new crystal engineering strategy is introduced in which host matrices are rationally designed to accommodate a predefined class of dopants and promote favorable host–guest interactions. This tailored-dopant matrix concept is demonstrated for dopant-induced organic room-temperature phosphorescence (RTP) using carbazole-based matrices and benzoindole dopants, a prototypical RTP system. Guided by the hypothesis that a herringbone packing of carbazole units promotes synergistic structural interactions with the dopant, a multiscale theoretical methodology is first developed to elucidate the intermolecular interactions stabilizing this motif in pristine carbazole crystals. These insights enable the design and synthesis of new host architectures exhibiting the targeted packing arrangement. Ultimately, the resulting single-crystalline host-guest materials exhibit long-lived organic RTP, with phosphorescence lifetimes of several hundred milliseconds. This work could establish dopant-tailored crystal engineering as a potential new paradigm for designing functional doped organic semiconductor crystals with tailored optoelectronic properties.

Table of Contents

I. Experimental section.	p.3

II. Synthesis.	p.6

III. Single crystals X-Ray diffraction analysis.	p.12

1) Crystal data and structure refinement.	p.12

2) Conformation and crystal structures	p.16

IV. Photophysical properties.	p.23

References.	p.28

**I. Experimental section**

***Reagents and materials***

Unless otherwise stated, all reagents used in the experiments were purchased from commercial sources without further purification.

***Experimental methods***

Nuclear magnetic resonance (^1^H and ^13^C) spectra were obtained on a Bruker Ultra Shield 300 MHz spectrometer for CDCl_3_. Chemical shift was relative to tetramethylsilane (TMS) as the internal standard. Resonance patterns are reported with the notation s (singlet), d (double), t (triplet), and m (multiplet).

High-resolution mass spectrometry was performed with an LC-MS (Agilent) spectrometer with electrospray as ionization (ESI) source. Chromatographic analysis was performed by an LC-MS system (Agilent, USA), an Agilent 1290 Infinity Binary LC with UV–vis photodiode array detector (DAD G4212A), coupled with an Agilent 6530 Accurate-Mass Quadrupole Time-of-Flight with a dual spray ESI source. G1316C Thermostatted column was set at 45 °C and two channel of elution solvents were water (A) and methanol (B), with 0.1% in volume of formic acid, respectively. Flow rate was 0.350 mL/min, injection volume was 20.00 μL (100 ppm), and column pressure was 600 bar as source parameters. The eluent conditions were (1) from 0 to 2 min with 10% B for isocratic condition, (2) from 10% to 100% B for 8 min for gradient condition, (3) 100% B for 3 min for isocratic condition, (4) from 100% to 90% B for 0.5 min for gradient condition, and (5) 90% B for 1.5 min for isocratic condition. Electrospray ionization mass spectrometric analysis (scan parameters) was performed in positive mode with nebulizer at temperature of 350 °C, flow of 10 L min−1, pressure of 45 psi, capillary voltage of 3500 V, fragmentor voltage of 135 V, skimmer voltage of 65 V, octopole RF peak of 750 V, and sheath gas temperature (295 °C) and flow (11 L min−1). A reference solution of the reference mass m/z 922.00979800 was used for continuous calibration. All data acquisition and analysis were managed by Agilent Mass Hunter Workstation Software (Agilent, USA).

Preparation of doped Cz single-crystals with Bd isomer (0.5 %): To a solution of Cz-based compound in methylene chloride (1 mL, 0.11x10-4 mol/L) at RT was added a solution of Bd-based dopant (0.5 mL, 0.04 g/L) in DCM. Then, an equivalent volume of *n*-hexane (1.5 mL) was slowly added and the biphasic mixture slowly evaporated until the complete formation of the crystals.

The crystal structures were determined by standard crystallographic methods (CCDC numbers of **OCz**, **OCz-*o*-Py**, **OCz-*m*-Py**, **Cz-Ph**, **Cz**, **Cz-*o*-Py**, and **Cz-*m*-Py**, are 2536511, 2536512, 2523899, 2536513, 2536586, 2020816, and 2020818, respectively. These data can be obtained free of charge from The Cambridge Crystallographic Data Centre via www.ccdc.cam.ac.uk/data_request/cif).

The data were collected at 223(2) K using a Bruker D8 Venture equipped with I*μ*S micro-focus sealed tube Mo Kα (*λ=* 0.71073 Å) and a PHOTON II 14 detector in Western Seoul Center of Korea Basic Science Institute. Data collection and integration were performed with APEX3 (Bruker, 2016) and SAINT-Plus (Bruker, 2016.^[1]^ Absorption correction was performed by multi-scan method implemented in SADABS.^[2]^ The structure was solved by direct methods and refined by full-matrix least-squares on *F*^2^ using SHELXTL program package (version 6.14).^[3]^ All the non-hydrogen atoms were refined anisotropically. The C-H H atoms were positioned with idealized geometry and were refined with fixed isotropic displacement parameters *U*_iso_(H) = 1.2 *U*_eq_(C) for aromatic and *U*_iso_(H) = 1.5 *U*_eq_(C) for methyl H atoms using a riding model.

Electrostatic potential calculations: The molecular geometries were optimized at the B3LYP/6-311++G(2d,2p) level of theory, as implemented in the Gaussian 09 quantum chemical package.^[4]^ The molecular electrostatic potential (MESP) pictures have been investigated using GaussView 6.1 software.^[5]^

NCI analysis computational details: The Gaussian16 code was used to optimize the geometries at the ground and excited states along with the range separated hybrid functional CAM-B3LYP.^[6]^ Structural optimizations and subsequent frequency calculations for both the ground and excited states were performed using an all electron Pople triple zeta basis set with one polarization function on all atoms and one diffuse function of heavier atoms, known as 6–311G(d,p), for H, C, N, O and Br atoms. The effect of the surrounding material was simulated by considering a Polarizable Continuum Model (PCM) of Tomasi and co-workers. Default radii (from the UFF, scaled by 1.1) were used and toluene was considered as solvent.

UV-vis absorption spectra at room temperature of the solutions were recorded on a Hitachi U-3310 spectrophotometer. Steady-state fluorescence spectra at RT of the solutions were obtained using a Varian Cary Eclipse Fluorescence Spectrophotometer.

Emission spectra in solution at 77K and in solid state (RT and 77K) were done using a Horiba Jobin-Yvon Fluorolog-3® spectrofluorimeter equipped with Hamamatsu R928 photomuliplier tube. Excitation were achieved with a 450 W continuous wave lamp (steady-state) or by pulse excitation with a FL-1040 UP xenon lamp (time-gated). Spectra were reference corrected for both the excitation source light intensity variation (lamp and grating) and the emission spectral response (detector and grating).

For 77K measurements, dilute solution in ethanol/methanol mixture (80/20 v/v) were prepared in a 5 mm diameter quartz tube fitted with a teflon valve. After the solution was degassed by sonication and argon bubbling, the tube was sealed. Phosphorescence spectra were recorded at 77 K in liquid Nitrogen using time-gated experiments.

Solid-state steady-state luminescence measurements were performed in 5 mm diameter quartz tubes with detection at an angle of 90°.

The lifetime and time-resolved emission spectra were recorded with a SPEX Fluorolog 3 spectrometer. The time-resolved photoexcitation source was from pulsed laser beam (343 nm, 4 mW) generated through a harmonic generator (Ultrafast Systems LLC, third harmonic) with a Pharos laser (Light Conversion, 1 kHz, 1,030 nm, 290 fs, divider 5600).

**II) Synthesis**

**1) Preparation of carbazole- and 2,7-dimethoxycarbazole-based matrices**

Matrices were prepared either from pure carbazole (prepared in a two steps sequence according to the procedure of Tian et al.^[7]^) or commercial carbazole, as well as from 2,7-dimethoxycarbazole recrystallized twice. The synthetic route involves through a two steps procedure combining (i) the preparation of the acetylenic carbazole precursor and (ii) its subsequent cross-coupling with the desired bromo-iodopyridine according to the following scheme.

**Scheme S1:** Two-step synthetic route for the preparation of the 2,7-dimethoxycarbazole-based matrices **OCz-*m*-Py** and **OCz-*o*-Py**.

The procedure for the synthesis of pure **Cz**, 9-H-propargyl-carbazole as well as the corresponding matrices and Bd-dopant are detailed in earlier reports.^[8]^

**Synthesis of 9-(3-(5-bromopyridin-3-yl)prop-2-yn-1-yl)-2,7-dimethoxy-9H-carbazole**

To a solution of 2,7-dimethoxycarbazole (0.34 g, 1.5 mmol) in DMF (10 mL) was added solid KOH (0.17 g, 3.0 mmol, 2 equiv) at 0 °C. After 20 min. stirring at this temperature, propargyl bromide (80 % solution in toluene – 0.33 mL, 3.0 mmol, 2 equiv) was added. The reaction was allowed to stir for a further 6 h at room temperature before water (20 mL) was added. The mixture was extracted with AcOEt (3 x 10 mL) and the combined organic layer dried over CaCl_2._ The solvent were removed under reduced pressure and the crude product chromatographed using EtOAc/*n*-hexane – 1/9 as eluent, yielding the desired product which was further purified upon recrystallization to give 2,7-dimethoxy-9-(prop-2-yn-1-yl)-9H-carbazole (246 mg, 62%) as white crystals.

**Sonogashira cross coupling of 2,7-dimethoxy-9-(prop-2-yn-1-yl)-9H-carbazole with corresponding bromo-iodopyridines: representative procedure for 9-(3-(5-bromopyridin-3-yl)prop-2-yn-1-yl)-2,7-dimethoxy-9H-carbazole (OCz-*m*-Py).**

Into a 100 mL two-necked flask were added Pd(PPh_3_)_2_Cl_2_ (0.06 g, 0.085 mmol) CuI (0.03 g, 0.17 mmol), 3-bromo-5-iodopyridine (0.48 g, 1.7 mmol), 2,7-dimethoxy-9-(prop-2-yn-1-yl)-9H-carbazole (0.45 g, 1.7 mmol) and triethylamine (8 mL) under a nitrogen atmosphere. After stirring at room temperature for 12 h, water (10 mL) was added and the mixture was extracted with AcOEt (3 x 10 mL). The combined organic layers were dried over CaCl_2_, and the solvents were removed under reduced pressure. The crude product was purified under column chromatography using CHCl_3_/*n*-hexane – 8/2 as eluent to afford the expected product as colorless needlelike crystals (0.61g 85%).^1^H NMR (300 MHz, CDCl_3_): 8.56 (d, *J=* 2.2 Hz,1H); 8.50 (d, *J=* 1.6 Hz, 1H); 7.88 (s, 1H) ; 7.85 (s, 1H); 7.78 (t, *J=* 2.0 Hz, 1H); 6.95 (d, *J=* 2.1 Hz, 2H); 6.88 (d, *J=* 2.2 Hz, 1H); 6.85 (d, *J=* 2.2 Hz, 1H); 5.17 (s, 2H); 3.94 (s, 6H).^13^C NMR (300 MHz, CDCl_3_): 158.41; 150.40; 141.27; 141.04; 120.85; 117.38; 93.72; 88.06; 79.32; 55.81; 33.03. HRMS (ESI), m/z: [M+H]: 421.05380, Calcd for C_22_H_17_BrN_2_O_2_: 420.0473. (error: 1.84 ppm).

**9-(3-(5-bromopyridin-2-yl)prop-2-yn-1-yl)-2,7-dimethoxy-9H-carbazole (OCz-*o*-Py)**

**OCz-*o*-Py** was obtained following a similar procedure than for **OCz-*m*-Py** using 5-bromo-2-iodopyridine. The compound was obtained as colorless needlelike crystals (0.58g 81%). ^1^H NMR (300 MHz, CDCl_3_): 8.62 (d, *J=* 2.4 Hz,1H); 7.89 (s, 1H) ; 7.86 (s, 1H); 7.73 (dd, *J=* 8.3, 2.0 Hz, 1H); 7.21 (d, *J=* 9.0 Hz, 1H); 6.98 (d, *J=* 2.1 Hz, 2H); 6.89 (d, *J=* 2.2 Hz, 1H); 6.86 (d, *J=* 2.2 Hz, 1H); 5.18 (s, 2H); 3.95 (s, 6H).^13^C NMR (300 MHz, CDCl_3_): 158.39; 151.15; 141.30; 140.81; 138.84; 128.25; 120.50; 117.30; 107.77; 93.61; 55.79; 33.05. HRMS (ESI), m/z: [M+H]: 421.05376, Calcd for C_22_H_17_BrN_2_O_2_: 420.0473. (error: 1.94 ppm).

**9-(3-(3-bromophenyl)prop-2-yn-1-yl)-9H-carbazole (Cz-Ph)**

A similar procedure was conducted using 3-Bromoiodobenzene. The product was purified under column chromatography using CHCl_3_/*n*-hexane – 5/5 as eluent to afford **Cz-Ph** as a white solid (0.67 g, 93%). Single crystals were obtained by slow evaporation of DCM/*n*-hexane mixture. ^1^H NMR (300 MHz, CDCl_3_): 8.11 (d, *J=* 7.8 Hz, 2H) ; 7.55-7.47 (m, 5H) ; 7.41-7.37 (m, 1H); 7.30-7.23 (m, 3H); 7.09 (t, *J=* 7.8 Hz, 1H); 5.25 (s, 2H). ^13^C NMR (300 MHz, CDCl_3_): 139.93, 134.52, 131.70, 130.38, 129.70, 125.97, 123.28, 120.51, 119.58, 108.80, 84.68, 82.44, 33.10.

OCz-*m*-Py

^1^H NMR spectrum of **OCz-*m*-Py** in CDCl_3_

^13^C NMR spectrum of **OCz-*m*-Py** in CDCl_3_

OCz-*o*-Py

^1^H NMR spectrum of **OCz-*o*-Py** in CDCl_3_

^13^C NMR spectrum of **OCz-*o*-Py** in CDCl_3_

Cz-Ph

^1^H NMR spectrum of **Cz-Ph** in CDCl_3_.

^13^C NMR spectrum of **Cz-Ph** in CDCl_3_.

**Table S1.** Names and structures of carbazole, 2,7-dimethoxy-9H-carbazole, previously reported parent matrices, dopants, and new daughter matrices.

| **Carbazole** |  |  |
| --- | --- | --- |
|  | **Cz** |  |
| **2,7-Dimethoxy-9H-carbazole** |  |  |
|  | **OCz** |  |
| **Previously reported parent matrices*** |  |  |
|  | **Cz-*o*-Py** | **Cz-*m*-Py** |
| **Dopants*** |  |  |
|  | **Bd-*o*-Py** | **Bd-*m*-Py** |
| **Daughter matrices** |  |  |
|  | **OCz-*o*-Py** | **OCz-*m*-Py** |
|  |  |  |
|  |  | **Cz-Ph** |

.

* C.Demangeat et al., *Advanced Optical Materials*, 2023, 11, 2300289.

**III. Single crystals X-Ray analysis.**

**1) Crystal data and structure refinement**

**Table S2.** **Crystal data and structure refinement for OCz.**

Identification code	**OCz**

Empirical formula	C14 H13 N O2

Formula weight	227.25

Temperature	223(2) K

Wavelength	0.71073 Å

Crystal system	Orthorhombic

Space group	P2**_1_**2**_1_**2**_1_**

Unit cell dimensions	a = 5.6570(9) Å	α= 90°.

b = 7.6123(13) Å	β= 90°.

c = 25.763(4) Å	γ = 90°.

Volume	1109.4(3) Å^3^

Z	4

Density (calculated)	1.361 Mg/m^3^

Absorption coefficient	0.091 mm^-1^

F(000)	480

Crystal size	0.379 x 0.212 x 0.191 mm^3^

Theta range for data collection	2.790 to 28.311°.

Index ranges	-7<=h<=7, -7<=k<=10, -34<=l<=34

Reflections collected	9345

Independent reflections	2741 [R(int) = 0.0249]

Completeness to theta = 25.242°	99.4 %

Absorption correction	Semi-empirical from equivalents

Max. and min. transmission	0.7457 and 0.6531

Refinement method	Full-matrix least-squares on F^2^

Data / restraints / parameters	2741 / 0 / 156

Goodness-of-fit on F^2^	1.062

Final R indices [I>2sigma(I)]	R1 = 0.0383, wR2 = 0.0970

R indices (all data)	R1 = 0.0413, wR2 = 0.0991

Absolute structure parameter	0.2(5)

Extinction coefficient	n/a

Largest diff. peak and hole	0.249 and -0.204 e.Å^-3^

**Table S3. Crystal data and structure refinement for OCz-*o*-Py.**

Identification code	**OCz-*o*-Py**

Empirical formula	C22 H17 Br N2 O2

Formula weight	421.28

Temperature	223(2) K

Wavelength	0.71073 Å

Crystal system	Monoclinic

Space group	P2**_1_**/c

Unit cell dimensions	a = 15.512(3) Å	α= 90°.

b = 5.9591(9) Å	β= 103.775(9)°.

c = 25.387(6) Å	γ = 90°.

Volume	2279.3(7) Å^3^

Z	4

Density (calculated)	1.228 Mg/m^3^

Absorption coefficient	1.819 mm^-1^

F(000)	856

Crystal size	0.324 x 0.162 x 0.104 mm^3^

Theta range for data collection	2.371 to 28.522°.

Index ranges	-20<=h<=20, -7<=k<=7, -34<=l<=34

Reflections collected	36102

Independent reflections	5724 [R(int) = 0.0403]

Completeness to theta = 25.242°	99.9 %

Absorption correction	Semi-empirical from equivalents

Max. and min. transmission	0.7457 and 0.6374

Refinement method	Full-matrix least-squares on F^2^

Data / restraints / parameters	5724 / 0 / 246

Goodness-of-fit on F^2^	1.036

Final R indices [I>2sigma(I)]	R1 = 0.0425, wR2 = 0.0974

R indices (all data)	R1 = 0.0744, wR2 = 0.1101

Extinction coefficient	n/a

Largest diff. peak and hole	0.495 and -0.610 e.Å^-3^

**Table S4.** Crystal data and structure refinement for **OCz-*m*-Py**.

Identification code	**OCz-*m*-Py**

Empirical formula	C22 H17 Br N2 O2

Formula weight	421.28

Temperature	223(2) K

Wavelength	0.71073 Å

Crystal system	Monoclinic

Space group	C2/c

Unit cell dimensions	a = 29.95(2) Å	α= 90°.

b = 4.094(3) Å	β= 110.37(2)°.

c = 31.31(2) Å	γ = 90°.

Volume	3600(4) Å^3^

Z	8

Density (calculated)	1.555 Mg/m^3^

Absorption coefficient	2.304 mm^-1^

F(000)	1712

Crystal size	0.185 x 0.071 x 0.050 mm^3^

Theta range for data collection	2.331 to 28.425°.

Index ranges	-39<=h<=40, -5<=k<=5, -41<=l<=41

Reflections collected	19462

Independent reflections	4530 [R(int) = 0.0889]

Completeness to theta = 25.242°	99.9 %

Absorption correction	Semi-empirical from equivalents

Max. and min. transmission	0.7457 and 0.6584

Refinement method	Full-matrix least-squares on F^2^

Data / restraints / parameters	4530 / 0 / 245

Goodness-of-fit on F^2^	0.993

Final R indices [I>2sigma(I)]	R1 = 0.0498, wR2 = 0.0896

R indices (all data)	R1 = 0.1321, wR2 = 0.1178

Extinction coefficient	n/a

Largest diff. peak and hole	0.315 and -0.320 e.Å^-3^

**Table S5.** Crystal data and structure refinement for **Cz-Ph**.

Identification code	**Cz-Ph**

Empirical formula	C21 H14 Br N

Formula weight	360.24

Temperature	223(2) K

Wavelength	0.71073 Å

Crystal system	Monoclinic

Space group	P2**_1_**/c

Unit cell dimensions	a = 18.0661(6) Å	α= 90°.

b = 5.5782(2) Å	β= 100.7858(13)°.

c = 16.3560(6) Å	γ = 90°.

Volume	1619.18(10) Å^3^

Z	4

Density (calculated)	1.478 Mg/m^3^

Absorption coefficient	2.537 mm^-1^

F(000)	728

Crystal size	0.471 x 0.128 x 0.127 mm^3^

Theta range for data collection	2.295 to 28.341°.

Index ranges	-24<=h<=24, -7<=k<=7, -21<=l<=21

Reflections collected	50855

Independent reflections	4034 [R(int) = 0.0410]

Completeness to theta = 25.242°	99.7 %

Absorption correction	Semi-empirical from equivalents

Max. and min. transmission	0.7457 and 0.5118

Refinement method	Full-matrix least-squares on F^2^

Data / restraints / parameters	4034 / 0 / 208

Goodness-of-fit on F^2^	1.056

Final R indices [I>2sigma(I)]	R1 = 0.0281, wR2 = 0.0679

R indices (all data)	R1 = 0.0441, wR2 = 0.0780

Extinction coefficient	n/a

Largest diff. peak and hole	0.256 and -0.461 e.Å^-3^

**b)**

*b*

*a*

**a)**

*a*

*b*

*0*

*c*

*b*

*0*

**c)**

**d)**

*d*_inter_

*θ*_herring_

*c*

*a*

**Figure S1. Crystal structures of Cz and OCz. a)** Molecular stacking of **Cz** along the *c*-axis. **b)** Molecular stacking of **OCz** along the *a*-axis. **c)** Arrays of **Cz** in the *a-b* plane. **d)** Arrays of **OCz** in the *b-c* plane. The blue arrows represent the molecular dipole moments.

**a)	b)**

*a*

*a*

*θ*_herring_

*d*_inter_

*b*

*b*

*H-bond*

*0*

*c*

*d*_pyr-pyr_

*c*

*a*

*a*

**Figure S2. Crystal structures of Cz-*o*-Py and CD 49. a)** Molecular stacking of **Cz-*o*-Py** along the *b*-axis. **b)** Arrays of **Cz-*o*-Py** in the *a-c* plane. **c)** Molecular stacking of **Cz-*m*-Py** along the *b*-axis. **d)** Arrays of **Cz-*m*-Py** in the *a-c* plane.

**b)**

***°***

***θ_2_=***

***θ_1_=***

***°***

***°***

***θ_2_=***

***°***

***θ_1_=***

**a)**

**Figure S3. Conformations of the molecules in the crystals. a) OCz-*o*-Py. b) Cz-*o*-Py**.

Angle *θ_1_* represents the torsion of the pyridine ring around the triple bond, while angle *θ_2_* is ascribed to the twist arising between the carbazole and the methylene spacer. Correspondingly, values close to ~60° for *θ_1_* and ~40° for *θ_2_* reflect that the structure is close to orthogonality.

**b)**

**a)**

***θ_2_=***

***θ_2_=***

***θ_1_=***

***θ_1_=***

**Figure S4. Conformations of the molecules in the crystals. a) OCz-*m*-Py b) Cz-*m*-Py**.

Angle *θ_1_* represents the torsion of the pyridine ring around the triple bond, while angle *θ_2_* is ascribed to the twist arising between the carbazole and the methylene spacer.

**b)**

**a)**

***θ_2_=***

***θ_2_=***

***θ_1_=***

***θ_1_=***

**Figure S5. Conformations of the molecules in the crystals. a) OCz-*m*-Py. b) OCz-*o*-Py**.

Angle *θ_1_* represents the torsion of the pyridine ring around the triple bond, while angle *θ_2_* is ascribed to the twist arising between the carbazole unit and the methylene spacer.

**a)	b)**

**Figure S6. Conformations of the molecules in the crystals. a) Cz-Ph. b) Cz-*m*-Py**.

Angle *θ_1_* represents the torsion of the benzene (**Cz-Ph**) and pyridine (**Cz-*m*-Py**) ring around the triple bond, while angle *θ_2_* is ascribed to the twist arising between the carbazole and the methylene spacer.

**a)**

**b)**

*a*

*0*

*c*

*0*

*θ*_herring_

*b*

*a*

**Figure S7. Crystal structure of Cz-Ph. a)** Molecular stacking of **Cz-Ph** phenyl subunit along the *b*-axis. **b)** Arrays of **Cz-Ph** phenyl subunit in the *a-c* plane.

**IV. Photophysical properties**.

**Parent matrices	Dopants	Daughter matrices**

**Figure S8.** Computed vertical transition energies at the CC2/Def2TZVP level using toluene as a PCM solvent. Different optimized geometries are considered: ground state (noted S_0_ geom.), first singlet excited state (noted S_1_ geom.) and first triplet excited state (noted T_1_ geom.).

**Figure S9. RTP decay curve @550nm of crystalline OCz-*o*-Py at 77K**

**Figure S10.** PL decay curve @550nm of crystalline OCz-*o*-Py at RT

**a)**

**b)	c)**

τ = 611 ms

**Figure S11. a)** RT steady-state PL spectrum of crystalline **Cz-Ph** doped with its isomer. **b)** PL decay @373 nm. **b)** PL decay @550 nm

**a)	b)**

**Figure S12. Pathways for charge transport a)** two dimensional (*e.g.* in **Cz**)**, b)** one-dimensional (*e.g.* **OCz-*o*-Py**)**.**

**Table S6: Examples of structurally similar (or impurity induced) host-guest crystalline RTP systems and their performances.**

1. SMART, SAINT-Plus and XPREP, Bruker AXS Inc., Madison, Wisconsin, USA, 2016.
2. G. M. Sheldrick, SADABS v 2.03, University of Göttingen, Germany, 2002.
3. SHELXTL v 6.10; Bruker AXS, Inc: Madison, Wisconsin, USA, 2000.
4. GaussView, Version 6.1, Roy Dennington, Todd A. Keith, and John M. Millam, Semichem Inc., Shawnee Mission, KS, 2016.
5. T. Yanai, D. P. Tew, N. C. Handy *Chem. Phys. Lett.* **393**, 51-57(2004).
6. Frisch MJ, Trucks GW, Schlegel HB, Scuseria GE, Robb MA, Cheeseman JR, Scalmani G, Barone V, Mennucci B, Petersson GA, Nakatsuji H, Caricato M, Li X, Hratchian HP, Izmaylov AF, Bloino J, Zheng G, Sonnenberg JL, Hada M, Ehara M, Toyota K, Fukuda R, Hasegawa J, Ishida M, Nakajima T, Honda Y, Kitao O, Nakai H, Vreven T, Montgomery JA Jr, Peralta JE, Ogliaro F, Bearpark M, Heyd JJ, Brothers E, Kudin KN, Staroverov VN, Kobayashi R, Normand J, Raghavachari K, Rendell A, Burant JC, Iyengar SS, Tomasi J, Cossi M, Rega N, Millam JM, Klene M, Knox JE, Cross JB, Bakken V, Adamo C, Jaramillo J, Gomperts R, Stratmann RE, Yazyev O, Austin AJ, Cammi R, Pomelli C, Ochterski JW, Martin RL, Morokuma K, Zakrzewski VG, Voth GA, Salvador P, Dannenberg JJ, Dapprich S, Daniels AD, Farkas Ö, Foresman JB, Ortiz JV, Cioslowski J, Fox DJ (2013) Gaussian 09 Revision D.01
7. X. Tian, L. Song, A.F.K. Hashmi, *Angew. Chem. Int. Ed.* ***59***, 12342-12346 (2020).
8. C. Demangeat, Y. Tang, Y. Dou, S. Dale, J. Z. Cielo, E. Kim, H.-J. Lee, A. D'Aléo, B. Hu, A.-J. Attias, Necessary and sufficient condition for organic room-temperature phosphorescence from host-guest doped crystalline systems, *Adv. Opt. Mater.* **11**, 2300289 (2023).
